# Periodontal tactile input activates the prefrontal cortex

**DOI:** 10.1038/srep36893

**Published:** 2016-11-11

**Authors:** Nobuaki Higaki, Takaharu Goto, Tetsuo Ichikawa

**Affiliations:** 1 Department of Oral and Maxillofacial Prosthodontics, Institute of Biomedical Sciences, Tokushima University Graduate School, 3-18-15 Kuramoto, Tokushima 770-8504, Japan

## Abstract

The prefrontal cortex (PFC) plays a role in complex cognitive behavioural planning, decision-making, and social behaviours. However, the effects of sensory integration during motor tasks on PFC activation have not been studied to date. Therefore, we investigated the effect of peripheral sensory information and external information on PFC activation using functional near-infrared spectroscopy (fNIRS). Cerebral blood flow (CBF) was increased around bilateral Brodmann areas 46 and 10 during visual and auditory information integration during an occlusal force (biting) task. After local anesthesia, CBF values were significantly decreased, but occlusal force was similar. In conclusion, the effects of peripheral sensory information from the periodontal ligament and external information have minimal impacts on occlusal force maintenance but are important for PFC activation.

The prefrontal cortex (PFC) is located in the anterior region of the cerebral cortex and plays a role in complex cognitive behavioural planning, decision-making, and social behaviors^1^,^2^,^3^. The contributions of the PFC to cognitive performance and working memory are a mechanism for active information maintenance as well as maintained information processing. A large number of neuroimaging studies have evaluated activation of the PFC using functional magnetic resonance imaging (fMRI) and functional near-infrared spectroscopy (fNIRS) in humans. PFC activation is reported to increase during working memory tasks such as the n-back task and the random number generation task^4^,^5^. The PFC can be divided into the dorsolateral prefrontal cortex (DLPFC), the orbitofrontal cortex, frontal pole, and the anterior cingulate cortex (ACC). The DLPFC and ACC are known to be activated in response to incongruent stimuli, consistent with a role in the implementation of cognitive control and performance monitoring^6^. Botvinick and colleagues reported that the ACC was activated in response to Stroop task stimuli, further relating its function to cognitive control^7^. Additionally, recent neuroimaging studies in patients with PFC-related dysfunction have informed the clinical significance of the PFC. Schizophrenic patients demonstrate impaired procedural learning as assessed by the Tower of Hanoi test^8^, and indeed it has been reported that schizophrenia patients show decreased PFC activation during working memory tasks relative to healthy controls^9^. Mah and colleagues reported that patients with lesions in the PFC show poor insight into their own deficits, and rely primarily on nonverbal cues to make inter personal judgments during the Interpersonal Perception Task^10^. Moreover, the relationship between decreased activation of the PFC and reductions in communication, social behaviour, affection, self-motivation, and cognitive function has been highlighted in dementia patients^11^. Kawashima *et al*. reported that learning tasks such as reading aloud and performing simple arithmetic might be useful for activating the PFC to prevent cognitive impariment^12^.

Activation of the PFC has been also discussed in the context of autokinetic tasks. Many brain regions are associated with autokinesis, such as the primary motor area, the prefrontal area, the supplementary motor area, the basal ganglia, and the cerebellum. Matsumura and colleagues reported that the PFC is more highly activated during the grasping task than the touch task using positron emission tomography (PET)^13^. Indeed, increased CBF in the PFC has been reported during toe flexion-extension movements^14^, shoulder flexion-extension movements^15^, and mouth opening and closing^16^. Regarding oral autokinesis, Narita and Otsuka reported that the simulated chewing task increases CBF in the PFC^17^,^18^. However, these previous studies only compared activation of the PFC between two different tasks, such as the finger relaxing and movement tasks or the foot and finger movement tasks. It is possible that different movement tasks have different degrees of impact on CBF in the PFC. Therefore, a movement task with the elimination of peripheral sensory information should be utilized to evaluate the intrinsic effect of peripheral sensory information on the PFC. However, the relationship between peripheral sensory information and activation of the PFC has not yet been evaluated. Further, there are few reports on the effect of sensory input during a motor task on PFC activation.

Therefore, we studied the PFC by focusing on oral movements and periodontal tactile sensation, which is easy to eliminate during a motor task. The effect of peripheral sensory information and external information on PFC activation was investigated by asking subjects to maintain occlusal force (periodontal tactile input) with the guidance of visual and auditory information.

## Results

**Figure 1:**
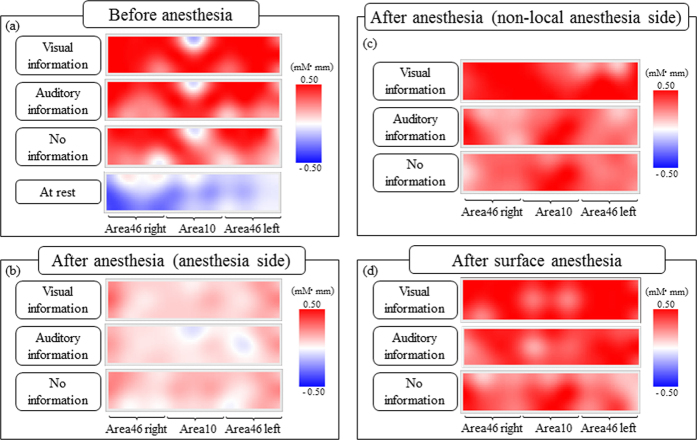
(**a**) Typical topographical patterns of cerebral blood flow (CBF) in the prefrontal cortex (PFC) before anesthesia. (**b**) Typical topographical patterns of CBF in the PFC after local anesthesia. (**c**) Typical topographical patterns of CBF in the PFC after anesthesia in biting in the contralateral side (non-local anesthesia side). (**d**) Typical topographical patterns of CBF in the PFC after surface anesthesia.

Figure 1 shows the typical topographical CBF patterns in the PFC upon task completion. Red and blue indicate CBF increases and decreases, respectively. In the task before local anesthesia, a strong red colour was observed in almost all regions, but a weak blue colour was identified in part of area 10 (Fig. 1a). No red was observed at rest, and blue was observed in areas 10 to 46 in the right hemisphere. After local anesthesia, diffuse red colouring was observed in almost all regions (Fig. 1b). The topographical patterns in the contralateral side (no local anesthesia) during the task after local anesthesia, as well as in the task after only surface anesthesia, were similar to those in the task before local anesthesia (Fig. 1c,d). Both were different from the patterns observed in the ipsilateral side after anesthesia.

**Figure 2:**
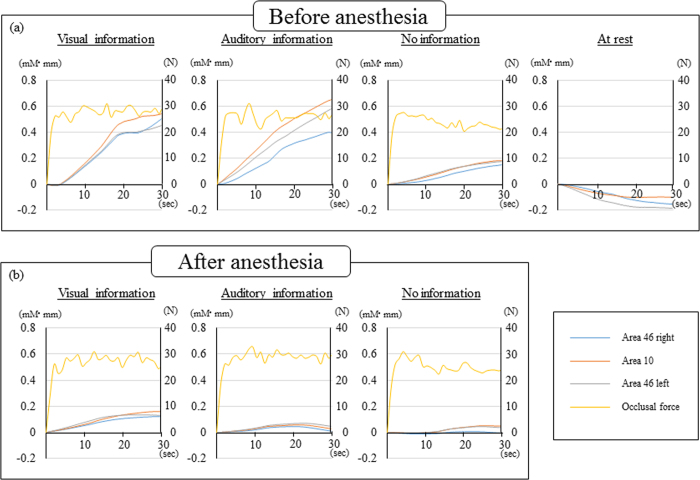
(**a**) Typical temporal patterns of CBF and occlusal force before local anesthesia. (**b**) Typical temporal patterns of CBF and occlusal force after local anesthesia.

Figure 2 shows typical temporal patterns of CBF and occlusal force during the 30-second task. Before local anesthesia, CBF patterns in the three experimental tasks (visual information only, auditory information only, no external information) monotonically increased around bilateral areas 46 and 10, and occlusal force was maintained at a constant value. Conversely, CBF at rest was decreased. Occlusal force in the visual information only and auditory information only tasks was controlled within an approximately constant range. In the task with without external information, occlusal force gradually decreased to suppress fluctuations. All subjects showed similar temporal patterns.

After local anesthesia, CBF increased slightly in the visual information only task, slightly increased and then decreased in the auditory only information task, and was unchanged in the no external information task. Temporal patterns of occlusal force were almost the same as those obtained before local anesthesia.

**Figure 3:**
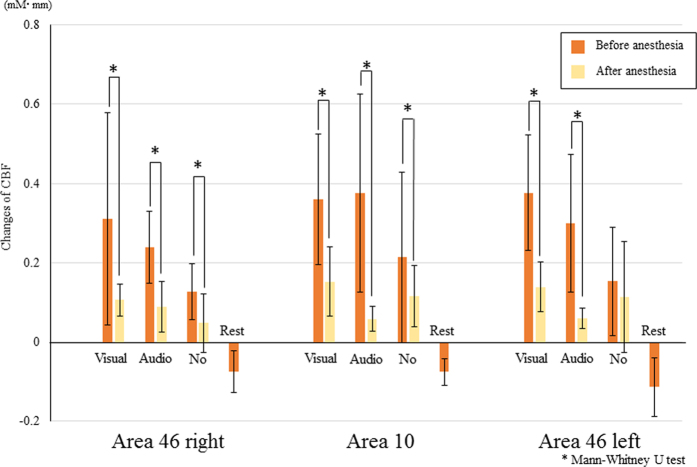
Changes in regional CBF before and after local anesthesia.

Figure 3 shows regional CBF changes upon task completion before and after local anesthesia. Regional CBF values were calculated by averaging probe data from each area: probes 7, 8, and 9 for area 46 left; probes 11, 12, 13, and 14 for area 10; and probes 15, 16, and 17 for area 46 right. Regional CBF values after local anesthesia were significantly decreased relative to those before local anesthesia in every area and experimental task, and this effect was particularly pronounced in the task with both visual and auditory information. Regional CBF values with visual information were higher than those with auditory information and without external information in every area before local anesthesia. A significant difference was found between visual and auditory information in area 46 right. After local anesthesia, no significant differences were observed between any areas.

**Figure 4:**
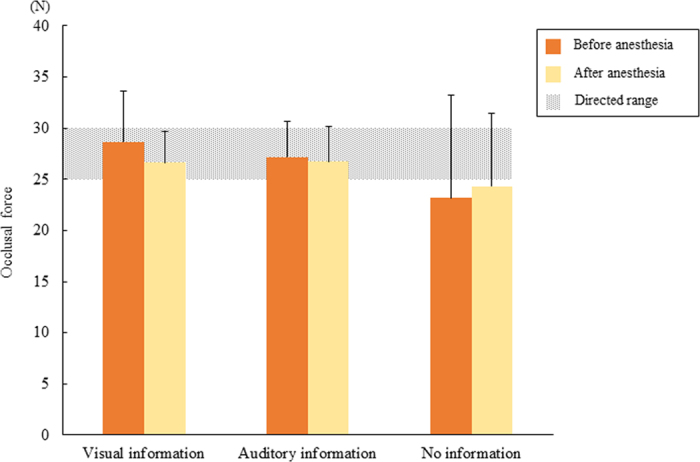
Mean occlusal force from 5 to 30 seconds after task onset.

Figure 4 shows mean occlusal force values from 5 to 30 seconds after task onset in the three experimental tasks. Occlusal force in the visual information only and auditory information only tasks was controlled within the directed range regardless of anesthesia, but it was below the directed range in the no external information task. No significant differences were found before or after local anesthesia with or without external information. Regarding the occlusal force, no significant differences were found within the external information before or after local anesthesia.

**Figure 5:**
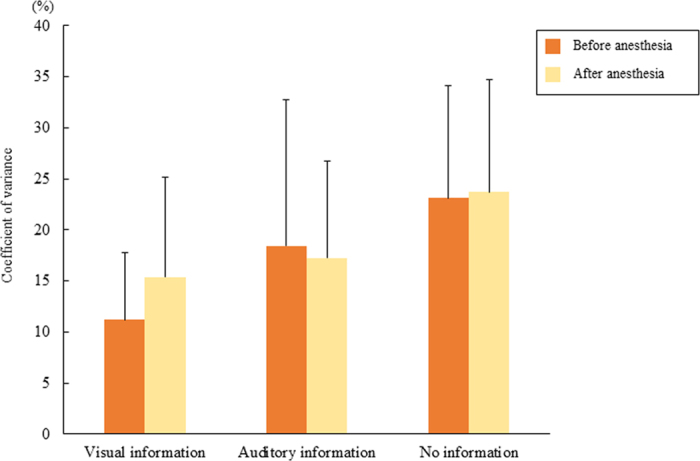
Coefficient of variation of occlusal force from 5 to 30 seconds after task onset.

Figure 5 shows the coefficient of variance of occlusal force in the three experimental tasks. The coefficient of variance in the visual information only task was lowest, and that in the no external information task was highest. However, no significant differences were found before or after local anesthesia with or without external information. Regarding the coefficient of variation of occlusal force, no significant differences were found within the external information before or after local anesthesia.

## Discussion

The goal of this study was to evaluate PFC activation in response to periodontal tactile and sensory integration using fNIRS. Non-invasive clinical tests of brain activation, including electroencephalography, magnetoencephalography, fMRI, PET, and fNIRS, have recently been employed in various clinical practices^19^,^20^,^21^,^22^,^23^,^24^. fNIRS offers the advantageous ability to continuously detect real-time functional hemodynamic responses as surrogates of brain activation during various tasks, without requiring motion restriction of the head or body.

In the present study, subjects were asked to maintain occlusal force between the upper and lower first molars for 30 seconds. A directed occlusal force of 25–30 N in this study was comparatively smaller than the maximum biting force of approximately 500 N^25^ and could be accomplished without muscle fatigue over the course of the experiment. The maintenance of occlusal force requires sensory information from the periodontal ligament, the muscle spindles of the jaw-closing muscles, the deep sensibility of the temporomandibular joint, and contact sensation of the tongue and lips^26^. However, the use of a small occlusal force at an interocclusal distance of 5 mm in the present study allowed the feedback of sensory information to primarily depend on the mechanoreceptors of the periodontal ligament^27^. Therefore, xylocaine anesthesia around the upper and lower first molar regions blocked afferent tactile sensation in the periodontal ligament and provided a near-complete elimination of sensory feedback in our study. When the influence of periodontal tactile loss due to local anesthesia on the PFC is discussed, one should consider the side effect of local anesthesia (experimental condition differences before and after local anesthesia). In the present study, local anesthesia was mainly injection into the periodontal ligament; influence on the surrounding tissues was minimal. The local injection causes tactile loss around the tooth but never inhibits gnathological sensation of the masseter and temporal muscles. This effect is also explained by the anatomical structure including the nerve tract. Activation of the muscles that open/close the jaw was evaluated before and after local anesthesia in a previous study; the authors found that activation of the temporal muscle (near the scalp and forehead) was not altered after anesthesia^28^. In fact, we found that the occlusal force was not changed after local anesthesia. The NIRS measurement site was distal from the local anesthesia region, and only slight tactile sensation loss in the buccal, lingual, and labial mucosa around the tooth due to the local anesthesia was observed. Still, it is possible that this could alter scalp or facial movements around the NIRS measurement site. The CBF patterns in the task after surface anesthesia only were similar to those in the task before local anesthesia, supporting the possibility that periodontal tactile loss led to decreased CBF in the PFC. We have also confirmed that the CBF in the PFC decreased after local anesthesia, while it increased during the task on the contralateral side. This makes it unlikely that a factor except periodontal tactile loss due to local anesthesia explains the CBF decrease in the PFC.

Visual and auditory information were used as external sensory information. A large number of studies have reported on the relationship between motor tasks and sensory feedback since the development of non-invasive clinical imaging methods^29^,^30^. Mishkin and colleagues reported that the sensorimotor area, occipital lobe, parietal lobe, and frontal lobe are activated by the visual feedback information during autokinetic tasks^31^. Ronsse *et al*. reported that the subjects with auditory feedback during motor tasks exhibit the activation of the sensorimotor area, the temporal lobe, the supplementary motor area, and the cerebellum^32^. Neurofeedback training (NFBT), which uses real-time visualizations of brain activity to direct the training of brain function, has recently received research attention. Johnston and colleagues reported that brain networks associated with specific emotions can be regulated by NFBT^33^. Despite these studies, no information is available about temporal changes in PFC activation during autokinetic tasks with sensory feedback integration.

Before local anesthesia, CBF was significantly increased by the presence of visual and auditory information during the occlusal force task. CBF with visual information was higher than that with auditory information. In humans, it is thought that visual sensation accounts for approximately 80% of sensory information input from the external environment, and external information based on visual sensation is important for motor learning^34^. Ronsse also reported augmented motor task performance after training with visual information compared to auditory information^32^. These results support the findings of the present study.

CBF changes in area 46 right, area 46 left, and area 10 showed similar characteristics with slow increase/decrease trends in response to each task. The localization of activity in these three PFC areas was not elicited by biting the transducer on the left side. Although the activation of area 46 has been associated with information selection, information extraction from long-term memory, and sensory integration^35^, the specific role of each PFC area, particularly area 10, remains unclear. The results of the present study suggest that the PFC areas work together to integrate sensory feedback during autokinetic tasks.

CBF values in the PFC in response to maintained occlusal force (biting) were dramatically decreased after local anesthesia. Although the relationship between peripheral sensory information and PFC activation has been investigated in previous fMRI studies^36^,^37^, few reports have validly addressed this relationship. Because the PFC is activated during decision-making in response to any motor task, it is difficult to design an appropriate control motor task for comparison. Additionally, while it is well known that periodontal tactile input is processed by the central pattern generator in the brainstem or by the cortical masticatory area located in the precentral gyrus, and that autokinetic masticatory movements are regulated by sensory input to the peripheral organ^38^, no study to date has demonstrated that intrinsic periodontal tactile input results in PFC activation. In this study, local anesthesia was used to eliminate the influence of decision-making during a motor task on PFC activation so that the intrinsic influence of periodontal tactile input on PFC activation could be evaluated. Thus, our experimental design allowed the clarification of the relationship between PFC activation and peripheral sensory information in a motor task.

In the presence or absence of peripheral sensory information, no significant differences in the maintenance of occlusal force were observed before or after local anesthesia, although the occlusal force value and its coefficient of variation were more stable before local anesthesia. It has been reported that the central pattern generator of masticatory movement dictates occlusal force during mastication by regulating mandibular movement via the supplementary and secondary motor areas^38^. The results in this study may indicate that occlusal force adjustments are generally performed in an unconscious feedforward manner regardless of the presence or absence of peripheral sensory information. This finding has also been implicated by the clinical use of dental implants, which are useful without connection to sensory tissue such as the periodontal ligament^39^,^40^. Conversely, PFC processing of periodontal tactile stimuli from the teeth may play a role in the prevention of cognitive impairment, as shown by recent clinical research in which dementia and cognitive impairment were associated with tooth loss^41^,^42^,^43^.

In conclusion, peripheral sensory information from the periodontal ligament and external information from the environment have minimal effects on autokinetic task outcomes; however, sensory and external feedback are quite important for PFC activation.

## Methods

### Subjects

Five young healthy Japanese subjects (one female and four males, mean age 26.0 ± 1.41 years) from Tokushima University of Japan volunteered to participate in this study. All subjects had normal dentition without any stomatognathic or central nervous system complications. The experimental overview and tasks were explained to subjects, and written informed consent was obtained. Data collection occurred over two days for each subject, and each 1-hour experiment was conducted in the evening. This study was conducted with the approval of the Ethics Committee of the Tokushima University Hospital (No. 1780) and all experiments were carried out in accordance with the approved guidelines.

### Activation of the PFC

PFC activation was evaluated in unrestrained patients using a wearable fNIRS device (WOT-100, HITACHI, Tokyo, Japan). This device measured relative changes in the concentration of oxy-haemoglobin by using light attenuation at two wavelengths of 705 and 830 nm, which easily pass through skin, tissue, and bone. Ten measuring probes of 16 total probes with a spacing interval of 30 mm covered three measurement areas located around the frontal pole: area 10 and the dorsolateral PFC, the right side of area 46, and the left side of area 46 (Fig. 1).

### Measurement of occlusal force

**Figure 6:**
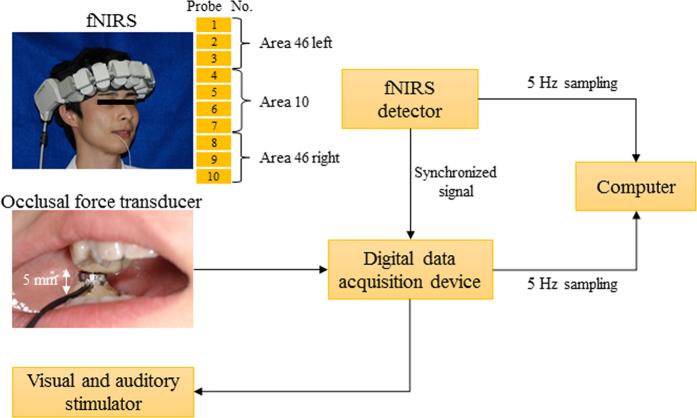
Description of the measurement apparatus.

Subjects were asked to bite an occlusal force transducer and maintain a given strength value. The occlusal force transducer was modified using an auto-curing acrylic resin: a commercial load cell (UNCDW-200N, Unipulse Corporation, Tokyo, Japan) was fitted to the occlusal surface to accommodate the bite of the upper and lower first molars. The interocclusal distance was 5 mm. Signals from the occlusal force transducer were digitized at a rate of 5 Hz with 14-bit accuracy using a digital data acquisition device (F372A, Unipulse Corp., Tokyo, Japan) and transferred to a computer (Fig. 6).

### Task

The experimental task was defined as maintaining occlusal force. Measurements were conducted in a soundproofed room. Any external disturbances were minimized, and room temperature and lighting were controlled. The subjects were seated on a chair in a relaxed manner and asked to continuously bite the occlusal force transducer with a force of 27.5 ± 2.5 N for 30 seconds.

### External information guiding occlusal force adjustments

**Figure 7:**
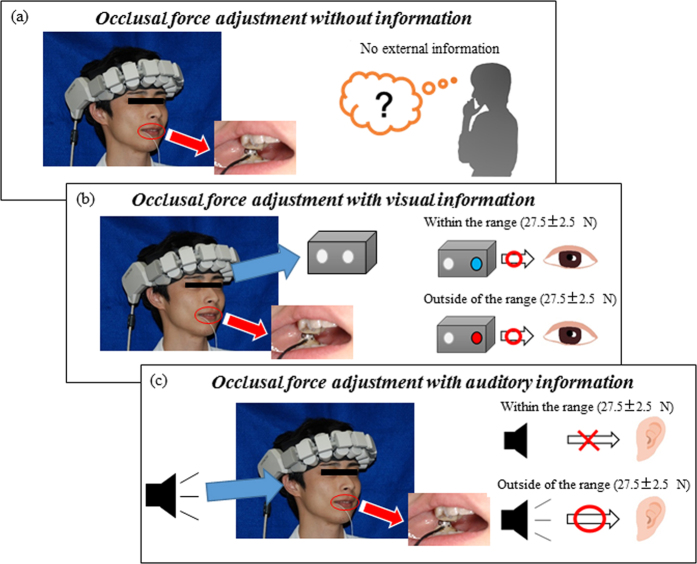
Three experimental tasks: occlusal force adjustment without information (**a**), occlusal force adjustment with visual information (**b**), and occlusal force adjustment with auditory information (**c**).

Visual and auditory information were provided to guide occlusal force adjustments (Fig. 7). When the measured occlusal force value was within or outside of the directed range, green LED and red LED lights were illuminated, respectively. When the measured occlusal force value was within or outside of the directed range, a buzzer also turned off and on, respectively. The apparatuses for visual and auditory information were self-designed and self-constructed.

### Measurement protocol

**Figure 8:**
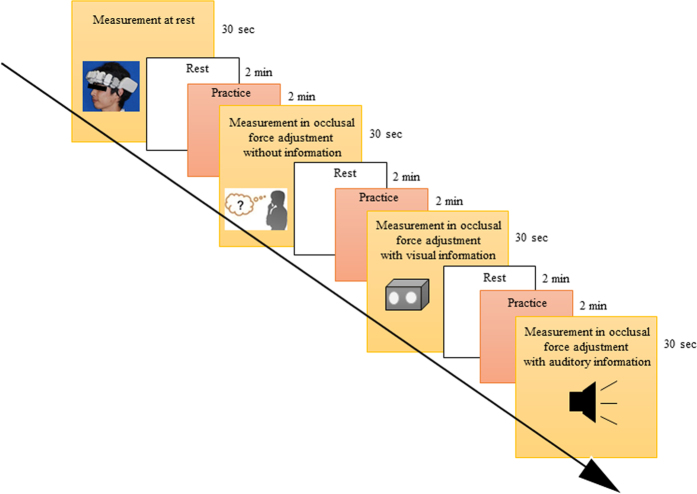
Serial measurement of four conditions including three experimental tasks. The order of experimental tasks was randomized in the serial measurement and measurements were repeated four times.

Figure 8 shows the experimental protocol. The following four measurement conditions including three experimental tasks were used: occlusal force adjustment with visual information, occlusal force adjustment with auditory information, occlusal force adjustment without external information, and measurement at rest. One course of measurement consisted of three phases: rest without a task for 2 minutes, practice for 2 minutes, and measurement for 30 seconds. In the practice phase, subjects were allowed to practice biting the transducer within the range of 27.5 ± 2.5 N using visual and auditory indicators. Measurement conditions were serially randomized and repeated four times per day. To examine the influence of periodontal tactile sensation on PFC activity, xylocaine (ORA Inj. Dental Cartridge 1.8 m, Showa Yakuhin Kako Co., Ltd. Tokyo, Japan) local anesthesia was administered to the upper and lower first molar regions with a 31 G needle (TERUMO, Tokyo, Japan). Measurements after local anesthesia were performed identically to those before local anesthesia.

To examine the effect of local anesthesia on PFC activity, the preliminary measurements in the contralateral side during the task were made after both local anesthesia and surface anesthesia only (Neozalocain Pasta^®^, Neo Dental Chemical Products, Osaka, Japan).

### Analysis

Data from fNIRS and the occlusal force transducer were synchronized and subsequently analysed (Fig. 1). The mean CBF for 10 seconds immediately after measurement onset was defined as a baseline and used to correct data obtained during the task. The period from 10 to 30 seconds after task onset was analysed to eliminate early adjustments made to maintain constant occlusal force. Following qualitative evaluation of the data, the mean and maximum values were analysed. One-way analysis of variance with Bonferroni post hoc tests was used to compare of CBF and occlusal force in the four conditions, and the Wilcoxon test was used to compare CBF and occlusal force before and after LA. All statistical analyses were conducted at a significance level of 0.05 with SPSS^®^ version 22.0 software (IBM Corp. Armonk, NY, USA).

## Additional Information

**How to cite this article**: Higaki, N. *et al*. Periodontal tactile input activates the prefrontal cortex. *Sci. Rep.* **6**, 36893; doi: 10.1038/srep36893 (2016).

**Publisher’s note:** Springer Nature remains neutral with regard to jurisdictional claims in published maps and institutional affiliations.
